# Recruiting Community Health Centers for Implementation Research: Challenges, Implications, and Potential Solutions

**DOI:** 10.1089/heq.2022.0195

**Published:** 2024-02-13

**Authors:** April Lee, Rachel Gold, Rachel Caskey, Sadia Haider, Teresa Schmidt, Emily Ott, Rinad S. Beidas, Amritha Bhat, William Pinnock, Melinda Vredevoogd, Tess Grover, Jena Wallander Gemkow, Ian M. Bennett

**Affiliations:** ^1^ OCHIN, Inc., Portland, Oregon, USA.; ^2^ Department of Science Programs, Center for Health Research, Kaiser Permanente, Portland, Oregon, USA.; ^3^ Departments of Medicine and Pediatrics, University of Illinois at Chicago, Chicago, Illinois, USA.; ^4^ Department of Obstetrics and Gynecology, Rush University Medical Center, Chicago, Illinois, USA.; ^5^ Department of Medical Social Sciences, Northwestern University Feinberg School of Medicine, Chicago, Illinois, USA.; ^6^ Department of Psychiatry and Behavioral Sciences, University of Washington, Seattle, Washington, USA.; ^7^ Health Services Research and Development Center of Innovation for Veteran-Centered and Value-Driven Care (HSR&D COIN), VA Puget Sound Health Care System, Seattle, Washington, USA.; ^8^ Health Research and Education Team, AllianceChicago, Chicago, Illinois, USA.; ^9^ Department of Family Medicine, University of Washington, Seattle, Washington, USA.

## Contributions to the Literature

- Under-resourced clinics (e.g., community health centers) wishing to participate in implementation research may be unable to do so because of financial and staffing resource limitations, and thus may be under-represented in implementation science results. This may introduce bias that limits study findings' generalizability, impacts evidence-based intervention adoption in similar care settings, and exacerbates health inequities.
- Recruitment bias is well studied in patient-randomized trials but not in clinic-randomized trials. This article presents two examples of studies seeking to recruit under-resourced clinics and highlights challenges inherent to recruiting such clinics in implementation science trials, and potential ramifications for study generalizability and health equity.

## Editorial

Implementation science generates knowledge about effective approaches for supporting the adoption and sustainment of evidence-based interventions (EBIs).^1^ Maximizing the external validity (and generalizability) of randomized trials' findings in implementation research requires recruiting study sites that represent the settings where adoption of a given EBI is desired.^2^ Recruitment to clinic-randomized trials may be challenging, however, when the care settings of interest are under-resourced clinics such as community health centers (CHCs), with the potential to exacerbate health inequities.

As the nation's health care safety net, CHCs serve >30 million socioeconomically vulnerable patients regardless of ability to pay, often operating with very limited financial and staffing resources.^3^ Achieving health equity will require optimizing EBI implementation in this setting.^4^ Yet although CHCs may recognize the value of taking part in EBI implementation trials, their resource limitations may prohibit study participation.^5^ This may result in such settings' under-representation in implementation research, introducing potential recruitment bias, weak evidence on EBI implementation in under-resourced settings,^6^ study findings that are not generalizable to the under-represented populations served by CHCs, and a threat that health inequities will be perpetuated.^6–8^

Recruitment bias in patient-randomized trials is well studied and known to adversely impact study findings' generalizability.^7^ As far less is known about recruitment bias in clinic-randomized trials, we present insights from two recent implementation studies targeting evidence-based perinatal care delivery in CHCs, and call for action to address this critical knowledge gap.

The Maternal Infant Dyad-Implementation (MInD-I) study, a clinic-randomized trial testing implementation of the Collaborative Care Model to treat perinatal depression in CHCs, aimed to recruit 20 primary care clinics from OCHIN's Practice-based Research Network, which at the time included 123 health systems across 19 states. Of 36 eligible clinics invited to participate, 67% (*n*=24) expressed initial interest in participation, but ultimately only 28% (*n*=10) agreed to participate.

Linking Inter-professional Newborn and Contraception Care (LINCC), a trial testing implementation of a model wherein postpartum contraceptive care is scheduled at newborn care visits, aimed to recruit 10 clinics affiliated with AllianceChicago, a Health Center Controlled Network including 45 safety-net clinics across 18 states at the time. Of 19 eligible clinics invited to participate, 89% (*n*=17) expressed initial interest in participation, but ultimately only 26% (*n*=5) agreed to participate. In both studies, clinics were asked to allocate protected time for multiple staff involved with implementation and study-related activities as a required component of participation. LINCC provided compensation to participating clinics; MInD-I did not.

In both trials, only half of the recruitment goal was achieved. Most sites that declined (85%; 64%) cited lack of capacity (e.g., staffing, time), competing clinical priorities, or insufficient leadership buy-in as the primary reason for not participating. One medical director noted, “We appreciate the concept of the performance improvement study and the clinical training offered; however, participation will require additional resources of various staff … that we do not have.” A second medical director noted, “We have multiple competing projects in flight right now and realize that we do not have the resources to make this project successful.”

## Health Equity Implications

The examples presented here demonstrate that even when CHCs are interested in trial participation, they may lack capacity to do so, especially if the targeted EBIs involve multiple complex implementation steps (e.g., intensive workflow changes). The costs of covering staff time to train and carry out practice change can be substantial,^8^ so when deciding on research participation, CHC leadership must balance the costs of diverting resources (e.g., loss of revenue for provider time) against the EBI's potential benefits and the feasibility of its post-study sustainment.

Here, many clinic leaders recognized the potential benefits of study participation, but ultimately declined to participate given resource limitations and concerns about impacts on clinic operations. This aligns with prior research showing that CHCs' research participation can be inhibited by resource constraints, including the inability to provide the up-front resources (e.g., personnel, time) often required for study engagement.^5,8,9^

Under-representation of CHCs in implementation science trials may threaten trial results' external validity, creating knowledge gaps about how to equitably disseminate EBIs in such settings. Ensuring that implementation research does not inadvertently widen health inequities in targeted outcomes in the communities served by these care settings will require understanding why and how under-resourced health care organizations participate in implementation-related trials.

Adding complexity, a tension in implementation science is that providing financial resources to help study sites implement an EBI may increase the feasibility of CHCs' study participation, but also lead to study results that do not reflect real-world implementation outcomes, as such implementation resources would not be available outside of a study context. Yet when studies do not provide resources to enable CHCs to participate, there is a risk that CHCs will be excluded from research, which could limit generalizability of study findings in these settings and exacerbate health inequities, analogous to how recruitment bias impacts patient-randomized trials.

## Solutions to Consider

These examples and prior research suggest some potential strategies for promoting CHC participation in implementation trials. Researchers might design studies with an equity lens, focus on interventions highly adaptable to under-resourced settings, use research funds to engage and compensate clinicians as constituents or co-investigators, and/or incorporate nonmonetary incentives (e.g., continuing medical education credits, electronic health record training).^4,6,9,10^

In recruitment, research teams might adopt flexible, multimodal outreach methods that build participant buy-in, and plan for a substantial investment of time and resources for recruitment activities.^9^ These recommendations should also be considered by funders, who could adjust funding models to enable clinician engagement as described above and address known research capacity gaps in under-resourced settings.

These recommendations are a starting point for engaging CHCs in implementation trials. Research is needed to better understand CHC-specific participation determinants, the implications of recruitment bias in clinic-randomized implementation studies, and strategies that address participation barriers without impacting results. As CHC patient populations are highly likely to benefit from effective EBI implementation, their inclusion in implementation research is essential to ensure the generalizability of study findings to these settings and their patients—and to improve the equitable implementation of EBIs.

## Consent for Publication

We obtained written permission to include the two medical directors' quotes noted in the Results section.

## Abbreviations Used

CHC: community health center
EBIs: evidence-based interventions
LINCC: Linking Inter-professional Newborn and Contraception Care
MInD-I: Maternal Infant Dyad-Implementation
